# Assessing organizational health literacy in hospitals by using the International Self-Assessment Tool for Organizational Health Literacy of Hospitals – a feasibility study in six European countries

**DOI:** 10.1186/s12913-025-13367-4

**Published:** 2025-10-01

**Authors:** Christa Straßmayr, Hanne Søberg Finbråten, Eva Maria Bitzer, Guglielmo Bonaccorsi, Maria Gabriella Cacciuttolo, Jan Dudra, Øystein Guttersrud, Zeynep Islertas, Celine Jeitani, Dušanka Krajnović, Christopher Le, Diane Levin-Zamir, Camilla Lombardo, Chiara Lorini, Benedetta Marcozzi, Luigi Palmieri, Denise Schütze, Maria Lucia Specchia, Alena Šteflová, Ivana Stević, Petr Struk, Brigid Unim, Stephan Van den Broucke, Patrizio Zanobini

**Affiliations:** 1 Competence Centre Health Promotion and Healthcare, Austrian National Public Health Institute, Stubenring 6, Vienna, 1010 Austria; 2 Department of Health and Nursing Sciences, Faculty of Social and Health Sciences, University of Inland Norway, P.O. Box 400, Elverum, 2418 Norway; 3 Department of Public Health & Health Education, University of Education Freiburg, Kunzenweg 21, 79117 Freiburg, Germany; 4 Department of Health Science, University of Florence, Viale GB Morgagni 48, Firenze, 50134 Italy; 5 Department of Life Sciences and Public Health, Catholic University of Sacred Heart, Largo Agostino Gemelli 8, Rome, 00168 Italy; 6 Klaudian Regional Hospital, tř. Václava Klementa 147/23, Mlada Boleslav, 29301 Czech Republic; 7 Norwegian Centre for Science Education, Faculty of Mathematics and Natural Sciences, University of Oslo, PO Box 1106, Blindern, Oslo, 0317 Norway; 8 Psychological Sciences Research Institute, Université Catholique de Louvain, 10 Place Cardinal Mercier, Bureau D215, Louvain-la-Neuve, 1348 Belgium; 9 Department of Social Pharmacy and Pharmaceutical Legislation, Faculty of Pharmacy, University of Belgrade, Vojvode Stepe 450, Belgrade, 11000 Serbia; 10 Department of Community Health, Norwegian Directorate of Health, Vitaminveien 4, Oslo, 0483 Norway; 11 School of Public Health, University of Haifa, 199 Aba Khoushy Ave. Mount Carmel, Haifa, 3103301 Israel; 12 Department of Life, Health and Environmental Sciences, University of L’Aquila, Palazzo Camponeschi, piazza Santa Margherita 2, L’Aquila, 67100 Italy; 13 Department of Cardiovascular, Endocrine-metabolic Diseases and Aging, Istituto Superiore di Sanità-ISS, Via Giano della Bella 34, Rome, 00161 Italy; 14 Czech Health Literacy Institute, Sokolská 490/31, Prague, 120 00 Czech Republic

**Keywords:** Organizational health literacy, Hospitals, Self-assessment, Health literate organization, Health literacy, Assessment tool, OHL-Hos, Feasibility, Heath literacy responsiveness, Health promoting hospitals

## Abstract

**Background:**

Hospitals can gain valuable insights into their current level of organizational health literacy (OHL) by using self-assessment tools. OHL self-assessment tools can serve as useful instruments for supporting the planning and implementation of OHL interventions aimed at promoting health equity and improving patient outcomes. This explorative study aimed to pilot the International Self-Assessment Tool for Organizational Health Literacy (Responsiveness) of Hospitals (OHL-Hos) among hospitals across six countries.

**Methods:**

The OHL-Hos, grounded in a comprehensive theoretical framework consisting of eight standards, 21 sub-standards and 141 indicators, was piloted in seven hospitals: one in Austria, Germany, the Czech Republic, Norway and Serbia, and two in Italy. In each hospital, the feasibility of using the OHL-Hos was investigated regarding acceptability, implementation, practicality, and integration, identifying strengths and areas for improvement using descriptive analyses. The self-assessment process included individual rating of an interdisciplinary and inter-hierarchical assessment team regarding OHL-Hos indicators from their personal perspectives, followed by a joint assessment to reach a consensus on different ratings. The process and experiences were documented in semi-structured forms, while the ratings on the indicators were documented numerically.

**Results:**

All hospitals successfully self-assessed their OHL, identifying strengths and areas for improvement. The self-assessment process varied slightly among countries. While the tool was considered important but lengthy and complex, introductory workshops facilitated successful implementation. The self-assessment process raised awareness and stimulated discussions on improving OHL, highlighting the tool’s potential for organizational development.

**Conclusions:**

The OHL-Hos can serve as a useful tool to identify strengths and areas for improvement in OHL in hospitals. The overall experience with the tool was positive and the joint assessment with the tool was found to foster consensus and enable reflection on OHL, but its comprehensive nature poses challenges to its implementation, leading to recommendations for developing a shortened version of the tool with simple language. Certain indicators require specific knowledge, suggesting different professional groups should address relevant parts.

**Supplementary Information:**

The online version contains supplementary material available at 10.1186/s12913-025-13367-4.

## Background

The growing appreciation of health literacy (HL) as a relational concept, implying that HL is the product of an individual’s capabilities and the HL-related demands and complexities of a system [1–3], has led to the development over the past two decades of concepts and tools that emphasize organizational settings for improving HL at the system level. Several terms, such as ‘health literate (health care) organizations’ [4], ‘organizational health literacy’ [5], ‘health literacy-friendly settings’ [6] and ‘organizational health literacy responsiveness’ [7] have been suggested to describe health literate environments, and several definitions have been proposed [8–10]. When focusing on hospitals/health care organizations, the definition of a health literate health care organization should be acknowledged, which states that ‘A health literate health care organization makes it easier for stakeholders (patients/relatives, staff/leadership, and citizens) to access, understand, appraise, and use/apply disease- and health-relevant information. It also strives to improve personal HL for making judgments and taking decisions in everyday life concerning health care (co-production), disease prevention, and health promotion to maintain or improve quality of life throughout the life course’ [9, 11]. According to the definition of the U.S. Department of Health and Human Services [12], the term ‘organizational health literacy’ (OHL) refers to the degree to which health care organizations equitably enable people, through organizational structures, policies, and processes, to find, understand, appraise, and use information and services to make health-related decisions and actions for themselves and others. Aligned with these definitions, a recently published scoping review [13] defines six criteria and attributes characterizing a health literate health care organization: 1. communication with service users, 2. easy access and navigation, 3. integration and prioritization of OHL, 4. assessment and organizational development, 5. engagement and support of service users, and 6. information and qualifications of staff. By reducing the organizational demands for people with limited HL, OHL interventions have the potential to promote health equity. Recent reviews suggest that OHL interventions contribute positively to patient-related outcomes, such as increased HL skills, participation in health care and increased self-management abilities [14, 15]. At the professional and organizational level, an increase in professionals’ competencies and practices regarding HL as well as organizational changes such as redesign of services to improve HL practices have also been mentioned as outcomes of OHL [14, 15]. Furthermore, OHL is considered a determinant of patient satisfaction [16].

OHL (self-)assessments tools are considered useful tools to support the planning and implementation of OHL interventions and have the advantage that they require minimal organizational resources [10]. Tools for OHL (self)-assessment are typically based on the principles of organizational development and quality management, and designed to collect data on the structures, processes, and culture of an organization. Relevant organizational characteristics are thereby defined as criteria or standards. The measurement of OHL characteristics is considered an important step in the quality cycle [17]. As such, a systematic approach to enhancing OHL should include a baseline assessment of current practices to detect gaps in existing OHL practice, inform the development of a flexible and detailed OHL action plan or strategy, and support an ongoing evaluation and monitoring of the progress towards establishing OHL within the organization [18].

In recent years, several tools have been developed to measure or assess OHL in health care settings. A recent scoping review [13] identified 17 tools to measure OHL. One of these is the Vienna tool of Health-Literate Hospitals and Healthcare Organizations (V-HLO-I). Farmanova et al. [10] recommended the V-HLO-I for its broad understanding of HL as the coproduction of health, quality, and safety and its roots in the settings approach of health promotion. The V-HLO-I was validated in its original German version [19], translated and culturally adapted into French [20] and piloted it in three Belgian hospitals [21]. The study team concluded that the tool is suitable to perform a needs assessment to increase the awareness of hospitals and to formulate targeted actions to further strengthen their HL responsiveness [21]. The V-HLO-I was improved and further developed into an international version by adapting it to different health care contexts on the basis of feedback received from different national contexts resulting in the International Self-Assessment Tool for Organizational Health Literacy (Responsiveness) of Hospitals (OHL-Hos) that was used in our study [22]. The OHL-Hos is intended to promote awareness and discussion of current OHL practice, to highlight what the main attributes of a health literate organization are, and to thereby stimulate an organizational self-learning process, identify strengths and areas for improvement of OHL, gain consensus for prioritizing HL interventions, and stimulate HL strategic planning. It also provides a benchmark for establishing OHL in hospitals in different health care systems [22]. Hence, the OHL-Hos could be considered a promising tool to support hospitals in their efforts to become health literate health care organizations.

To our knowledge, to date, the OHL-Hos has not been piloted in any country. Feasibility studies are recommended to evaluate the suitability and sustainability of new interventions prior to widespread implementation [23]. Due to the differences in the organization and funding of health services across European countries, and the aim of implementing the OHL-Hos in different national settings, a feasibility study was deemed necessary to determine the appropriateness and sustainability of the OHL-Hos and its self-assessment process. This article describes the results and experiences from six European countries in assessing both the feasibility of the tool and the self-assessment process.

## Methods

### Objectives and research questions

The objectives of the study were to pilot the national versions of the OHL-Hos in hospitals from six countries: Austria (AT), Czech Republic (CZ), Germany (DE), Italy (IT), Norway (NO) and Serbia (RS), and to explore the feasibility of implementing the tool. Specifically, four general areas of feasibility were investigated, as described by Bowen et al. [23]: acceptability, implementation, practicality, and integration. The research questions were:

- How do users experience the feasibility of the OHL-Hos and the self-assessment process (referring to acceptability, implementation, practicality and integration)?
- To what extent does the use of the OHL-Hos enable the identification of OHL strengths and areas for improvement?
- How does the use of the OHL-Hos support benchmarking among different national health care organizations?

While the first and second research questions were investigated empirically, the third question was only investigated theoretically in the discussion section.

### Material: the OHL-Hos

**Fig. 1:**
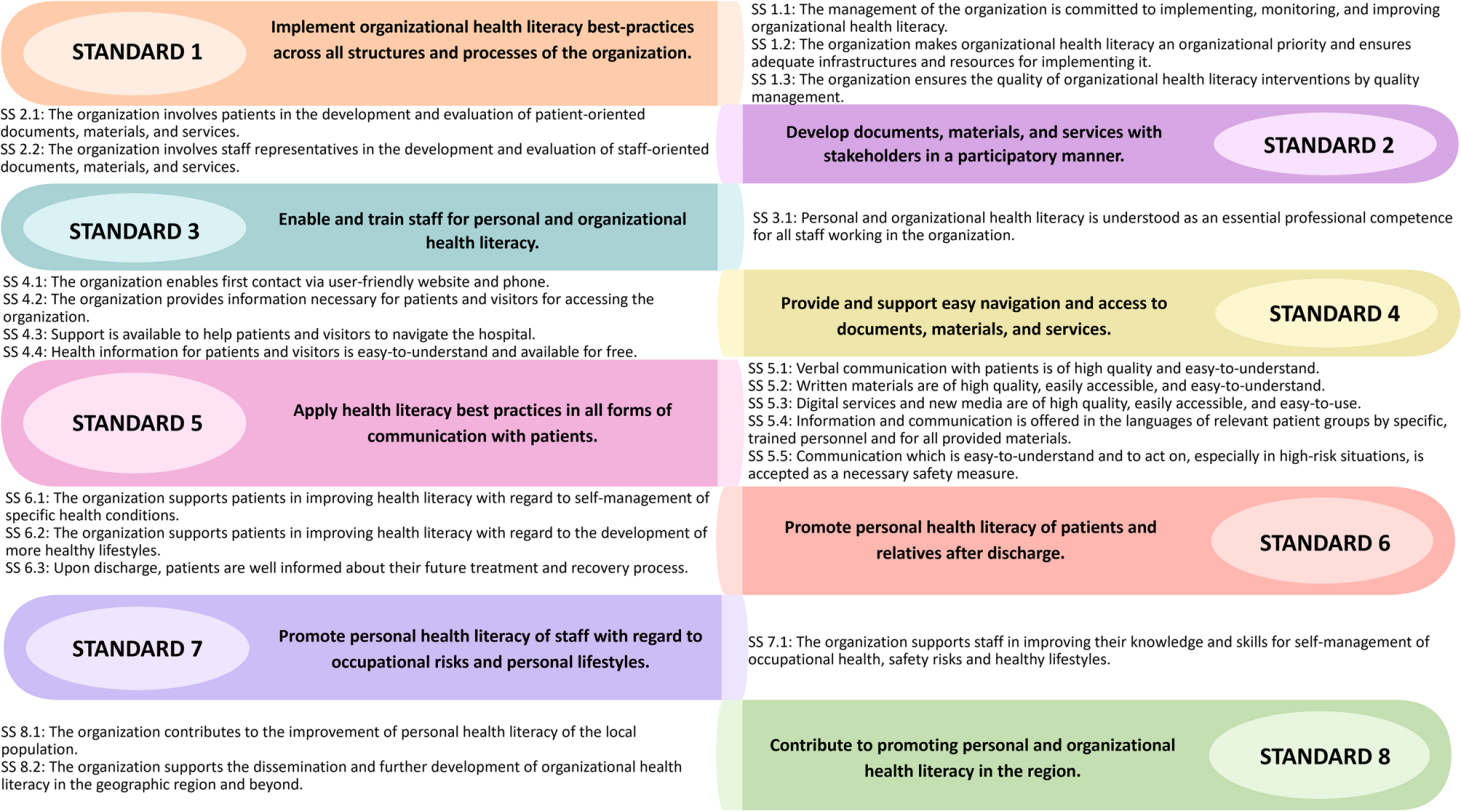
Standards and sub-standards (SS) of the International Self-Assessment Tool for Organizational Health Literacy of Hospitals

The OHL-Hos as used in this study is a modified version of the V-HLO-I. Like its predecessor, it is based on the Vienna concept as theoretical framework [19] and the definition of ‘health literate health care organization’ proposed by Pelikan and Dietscher [11]. The OHL-Hos acknowledges HL as a core concept of health promotion and relies on the health promoting settings approach. It is directed towards: (a) patients, (b) staff, (c) the resident population of the community a hospital serves, and (d) organizational structures and processes to implement the comprehensive OHL concept into the everyday practice of the organization across four domains (i) access to, living and working in the organization, (ii) diagnosis, treatment and care, (iii) disease management and prevention and (iv) healthy lifestyle development. Based on this framework the tool has eight standards and 21 sub-standards (Fig. 1).

The standards and sub-standards are operationalized by 155 indicators including sub-indicators, or 141 indicators without sub-indicators. The indicators operationalize concrete observable or measurable elements that are aligned with the principles for health care standards of the International Society for Quality in Health Care [24] and standards for health promotion in hospitals [25]. The underlying understanding of the earlier OHL debate in the United States of HL and OHL as a concept for improving the quality of health care services was considered, and accepted quality assurance methods in health care were explicitly applied [9, 11].

The OHL-hos is embedded in a comprehensive document with an introduction, background information on HL and OHL, and instructions on how to use the tool. Furthermore, a glossary and a template for an action plan are provided.

For each indicator, four categories indicating the degree of fulfillment are defined: completely fulfilled (76–100%) (‘yes’), fulfilled to a larger extent (51–75%) (‘rather yes’), fulfilled to a lesser extent (26–50%) (‘rather no’), or not fulfilled (0–25%) (‘no’). If a specific indicator is considered as not applicable (N/A) to the organization, it is categorized as such. For each indicator, the instrument offers additional space for comments that can explain or justify the self-assessment. The OHL-Hos is available at https://m-pohl.net/ReferencesOHL.

### The self- assessment process

**Fig. 2:**
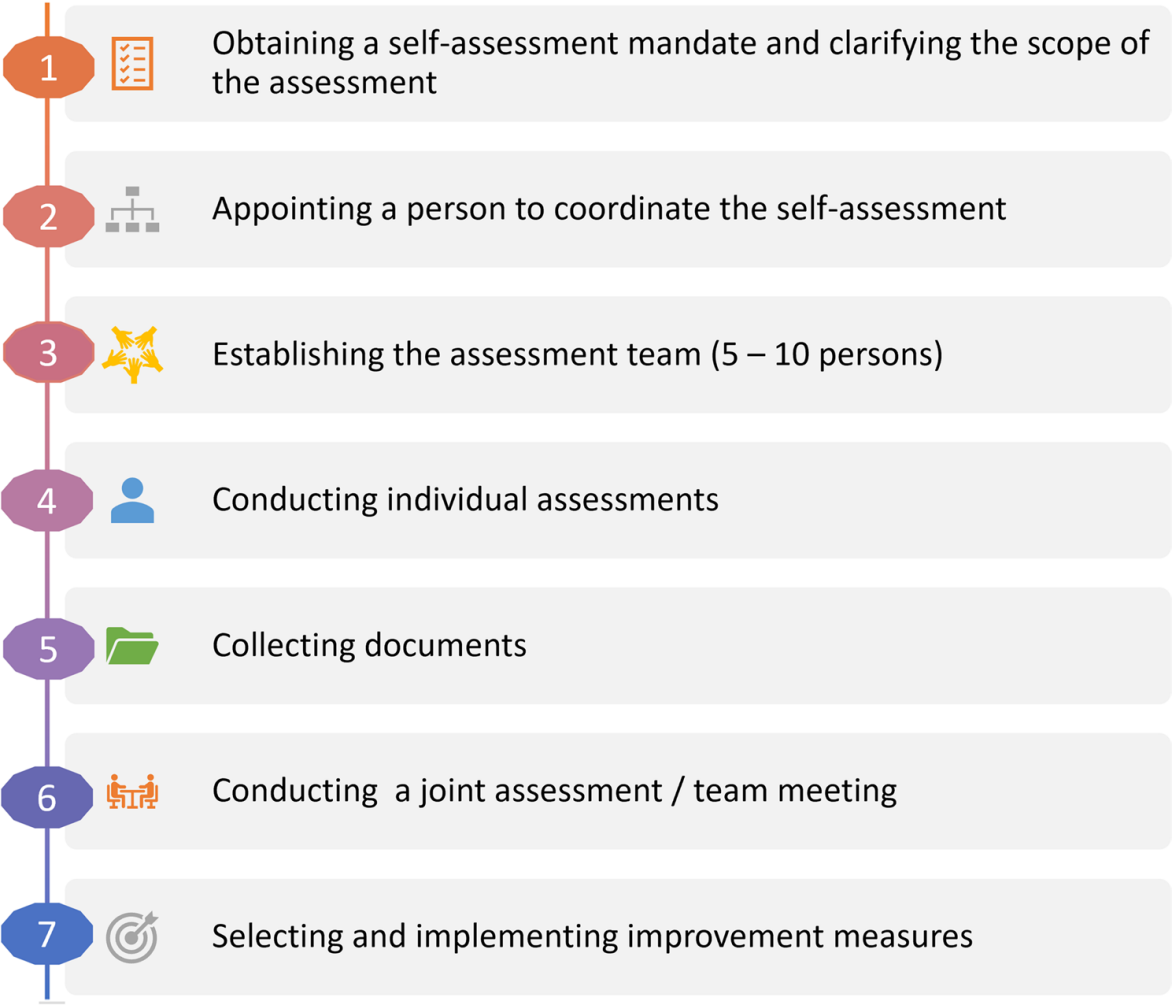
The seven steps of the OHL-Hos self-assessment process

The instructions on how to use the OHL-Hos [22] recommends seven steps (Fig. 2). It starts with obtaining a self-assessment mandate from the responsible management of the hospital after clarifying the scope of the assessment (step 1). Next, the management should appoint a hospital-internal person to coordinate the self-assessment (step 2). An assessment team of five to ten people, ideally from executive management, quality management, health promotion, human resource development, medicine, nursing, therapeutic professions, building service/maintenance, patient-ombudsman/woman, self-help groups and patient representatives, and communications/spokesperson should be established (step 3). Each member of the assessment team should then provide an individual assessment completing the tool from their personal perspective (step 4). The individual assessments of all team members should then be captured on one table (excel-sheet), for ease of comparison and then be discussed in the following joint assessment (team meeting). Collecting documents, where possible, is suggested to assess some of the indicators (these are indicated with * in the tool) (step 5). This step should be seen as a supplement to step 4 and should take place simultaneously. In a joint assessment, the different individual assessments are brought together (step 6). It is recommended that a moderator be appointed to facilitate the discussion. In preparation for the joint assessment (step 6), the first step is to identify indicators with similar ratings. Then, the focus should be on indicators with significantly different ratings. The latter should be clarified and discussed during the joint assessment, leading to a diagnosis of the strengths and weaknesses concerning OHL of the institution or of the specific unit. On this basis, areas can be defined for selecting and implementing measures to improve specific aspects of OHL (step 7).

A specific focus of this pilot study was on the feasibility of steps 3, 4, and 6 of the proposed self-assessment process, as inspired by the ‘RAND Appropriateness’ method [26]. According to this method, individual members of the internal multidisciplinary assessment team complete the questionnaire, after which the results are discussed in each service during a joint group assessment to achieve consensus. Step 5, collecting documents, was considered optional, and step 7 was considered beyond the scope of a pilot study.

### Data collection and data analyses

The study was conducted within the WHO Action Network on Measuring Population and Organizational Health Literacy (M-POHL) [27]. In each country, a national research team coordinated the study on the national level and closely cooperated with the hospital-internal coordinator(s) and/or the participants from the selected hospital(s). The OHL-Hos was translated into German, Czech, Italian, Norwegian and Serbian following a common protocol suggesting two forward translations followed by a meeting to reach consensus on to the most appropriate version. Similar types of difficulties and challenges in the translation process were experienced, and similar strategies were used to address them (e.g. replacing original terms which were imprecise when translated with more precise terms). Next, all language versions of the tool were culturally adapted to the national context of hospitals and health care systems. Thereafter, the OHL-Hos was piloted in seven hospitals in six countries (Table 1). All hospitals included are non-profit organizations, except for the Czech hospital, which could be considered both a for-profit and a non-profit organization. The hospitals included from CZ, DE, IT, NO and RS are all government owned either regionally or locally, while the participating hospital in AT is owned by a religious organization (non-governmental organization). Six out of the seven pilot hospitals offer both basic and continuing education/ongoing training for the staff. One of the Italian hospitals offers only basic training.

**Table 1:** Characteristics of the participating hospitals

Country							
	**AT**	**CZ**	**DE**	**IT-A**	**IT-B**	**NO**	**RS**
Type of hospital	general and acute care	general and acute care	general and acute care	general and acute care	general and acute care	general and acute care	specializing in addiction diseases
Basis for self-assessment	entire organization	entire organization	one unit (pediatrics)	entire organization	entire organization	management for the entire organization, staff at department level	entire organization
Location	urban	urban	large urban	urban	urban	large urban	metropolis
Staff: full-time equivalents	4500	1808	420	1674	1295	11,300	145

Urban: ≥15,000 and <100,000 inhabitants, large urban: ≥100,000 and < 1,000,000 inhabitants, metropolis: ≥1,000,000 inhabitants

*AT=*Austria, *CZ*=Czech Republic, *DE*=Germany, *IT-A*=Hospital A in Italy, *IT-B*=Hospital B in Italy, *NO*=Norway, RS=Serbia

Piloting was performed in AT from October 2019 to March 2020, in CZ from April 2023 to June 2023, in DE from February 2023 to June 2023, in IT from September 2023 to July 2024, in NO from October 2022 to November 2022 and in RS from December 2023 to July 2024. The different timing between the participating countries is due to the fact that the first attempt to conduct the study was made shortly before the onset of the Covid-19 pandemic, and that the study was suspended during the pandemic.

A reporting template was developed to qualitatively and quantitatively document the process, results and experiences of the piloting by the research teams. The template included descriptive questions regarding the hospital in which piloting was performed as well as questions for assessing the feasibility and usability of the tool. When more than one pilot took place in a country, the template had to be completed separately. For each pilot there was close cooperation between the research team and the hospital-internal coordinators. The feasibility of the piloting process was assessed through direct observation of the joint assessment and semi-structured interviews (see supplementary file 1 for the detailed questions) with the internal coordinators within the hospital and/or directly with the participants either by phone or in person.

The reporting template also included numerical data from the self-assessment. To allow descriptive analysis of the pilot data, a numerical score was attributed to each response category (3 = yes, fulfilled completely (76–100%); 2 = rather yes, fulfilled to a large extent (51–75%); 1 = rather no, fulfilled to a lesser extent (26–50%); 0 = no, not fulfilled (0–25%); N/A = indicator is not applicable). N/A responses were treated as missing values for the analysis. In the NO pilot, the labels for the response categories were used slightly differently (fulfilled to a very large extent (76–100%), fulfilled to a large extent (51–75%), fulfilled to some extent (26–50%) and fulfilled to a small extent (0–25%) as the shortened ‘rather yes’ and ‘rather no’ were not well accepted when pre-testing the translation.

Calculations were performed for each pilot hospital separately. For each indicator, sub-standard and standard, means and standard deviations were calculated across all participants. The mean value of a standard was calculated from the means of each indicator within the standard for all participants. Sub-indicators were equally weighted as one indicator in the mean of the standard. Each sub-standard was weighted equally in the overall mean (= mean of a standard), regardless of the number of indicators in the sub-standard.

Using the results from the joint assessment, means were categorized into three groups, indicating: (i) areas of strengths (mean ≥ 2.0); (ii) areas of the intermediate stage needing attention (mean > 1.0 and < 2.0); (iii) areas of weaknesses (needing attention, mean ≤ 1.0). Standard deviations were categorized, indicating consensus level: (i) high (*sd* ≤ 0.75), (ii) medium (0.75 < *sd* < 1.0), and (iii) low *sd* ≥ 1.0).

Inferential analyses were not conducted due to the small number of participants (*n* = 7–24) from each hospital and that the data originated from only one hospital in five countries and two hospitals in another country.

Data were anonymized after the joint assessment (step 6) and before submitting it to the research team. General Data Protection Regulations’ compliance was ensured by all countries.

## Results

### Feasibility of the tool

#### Acceptability

Regarding acceptability (i.e., how the intended individual recipients react to the intervention (i.e., to the self-assessment process and the tool)), all participating countries obtained a mandate from the hospital management to perform the self-assessment and appointed a hospital-internal coordinator. The assessment team for the individual and the joint assessment (range from seven participants in DE to 24 participants in RS for both individual and joint assessment) was selected and recruited by the hospital-internal coordinator, who had the task of putting together a team in such a way that as many perspectives as possible were covered. The composition of the assessment teams varied across the different hospitals, with teams comprising members from different disciplines and levels within the hospital hierarchy. Not all professional groups were represented in every hospital (Table 2).

**Table 2:** Participants in individual and joint assessments, and time spent on the assessments

Selected details on the assessment process	Country						
**Individual assessment**	**AT**	**CZ**	**DE** ^**#**^	**IT-A**	**IT-B**	**NO^**	**RS°**
Number of participants	11	10	7	10	9	12	24
Participants’ professional role:							
Management	3	3		2	2		1
Quality management	2	1		1		1	3
Health promotion	1		1	1		1	1
Human resource development	1	1	1	1		1	
Medicine	1	1	3	1		1	8
Nursing	1	1		1	2	4	8
Communications / spokesperson		1			1	1	
Physiotherapist						1	
Occupational therapist						1	
Other	2^i^	2^ii^	2^iii^	3^iv^	4^v^	1^vi^	3^vii^
Average duration of the assessment (in minutes)	120	180	^+^	330	210	105	204
**Joint assessment**							
Number of participants	7	10	7^#^	10	9	11	24
Participants’ professional role:							
Management	1	3		2	2		1
Quality management	2	1		1		1	3
Health promotion	1		1	1		1	1
Human resource development	1	1	1	1		1	
Medicine	1	1	3	1			8
Nursing	1	1		1	2	4	8
Communications / spokesperson		1			1	1	
Physiotherapist						1	
Occupational therapist						1	
Other		2^ii^	2^iii^	3^iv^	4^v^		3^vii^
Average duration of the assessment (in minutes)	240	360	60^#^	120	90	200	105

^i^patient management, ^ii^patient ombudsman/woman, epidemiologist, ^iii^therapeutic profession and laboratory technician, ^iv^therapeutic professions, building services engineering/maintenance, patient-ombudsman/woman, self-help and patient representatives,^v^residents of hygiene and preventive medicine, administrative staff ^vi^health worker, ^vii^therapeutic professions, ^#^only Standards 4, 5, and 6 were included in the joint assessment. ^Joint assessment was conducted for the management and health care professionals separately. **°**Serbia had four pilot groups based on specific roles in the hospital (group 1: included mainly health care associates who are not considered health care professionals by legislation but provide care together with health care professionals, group 2: mainly managerial capacities, group 3: nurses, and group 4: mainly medical doctors from different wards). ^+^Information not documented

*AT=*Austria, *CZ=*Czech Republic, *DE=*Germany, *IT-A=*Hospital A in Italy, *IT-B=*Hospital B in Italy, *NO=*Norway, *RS=*Serbia

Feedback from the internal coordinators and/or participants within the hospitals indicated that the topic of OHL was considered important, and that people were motivated to engage with the tool. However, concerns were raised about the complexity and length of the tool. In the German hospital a participant suggested that ‘*the tool must be shortened so that it is more manageable and does not have a negative impact on the motivation of the participants to complete the self-assessment and indicators must be optimized in terms of content and terminology’ (participant*,* DE).* Furthermore, the full assessment process was perceived to be time-consuming, resulting in some professionals refusing to participate. Yet, with regard to the learning experience on OHL, the joint assessment was considered highly valuable as it provided an opportunity to reflect on organizational practices and identify strengths and areas for improvement in the hospital’s OHL.

#### Implementation

Regarding implementation (i.e., the extent to which the intervention was fully implemented as planned and proposed), the individual and the joint assessments were conducted according to the protocol in the Austrian, Italian and Serbian hospitals, whereas some adaptations were made in other countries. In the Czech hospital, the joint assessment was conducted according to the protocol, but the individual assessments were not collected prior to the joint assessment and instead presented at the joint assessment meeting followed by a discussion. In the German hospital, individual assessments were conducted according to the study protocol, while for the joint assessment, indicators that were of most interest to the participants (Standards 4, 5 and 6) were selected for discussion. In the Norwegian hospital, the individual assessment was conducted according to the study protocol, except that the clinical staff did not respond to sub-standard 1.1 as the content could be considered most relevant for managers. The joint assessment in the Norwegian hospital was conducted separately for the management and clinical staff groups. In the Serbian hospital, the joint assessment was conducted for managerial capacity, nurses, medical doctors and health associates (i.e., therapeutic staff) in four separate groups. In AT, CZ, and RS, the joint assessments were moderated by the internal coordinator of the hospital and a member of the national research team. In the German and Norwegian hospitals, a member of the national research team moderated the joint assessment. In the Italian hospitals the moderation was done solely by the internal coordinator. The hospitals in AT, DE, NO, and RS held an introductory workshop for the assessment participants prior to the individual assessment in which some background information on the concept of OHL was provided as well as instruction as to how to complete the OHL-Hos.

#### Practicality

Regarding practicality (i.e., the extent to which the intervention can be delivered when resources, time or commitment are constrained in some way), the tool was considered lengthy and time-consuming to complete. The completion time for the individual assessments ranged from one hour and 45 minutes in the Norwegian hospital to five and a half hours in an Italian hospital. The time for the joint assessments ranged from one hour in the German hospital to six hours in the Czech hospital (Table 2). The tool could be either completed in a Microsoft Word or Excel version. In NO the clinical staff found the Excel format demanding and would prefer a format on a digital platform. One participant said that (s)he would prefer a print-friendly layout of the tool. In AT, where the Word version was used, it was reported by the hospital-internal coordinator that ”*50 pages were ’much too long*,* for management it is unrealistic to fill out such a long survey” (hospital-internal coordinator*,* AT)*.

As for the usability of the OHL-Hos, all countries reported that some indicators were repetitive and redundant, and that some were hard to understand due to demanding language and several unknown terms. The glossary and the introductory workshop were highly appreciated (NO, DE). The Czech and the Italian team considered the language of the tool to be clear and comprehensive. Some professional groups in AT, DE, IT, NO, and RS reported that they did not have sufficient knowledge or insight to respond to all indicators. This was especially true for the indicators of Standard 1, which refers to organizational structures and processes that were mostly considered top level management issues. In the Italian hospitals, Standard 8 was considered not relevant in a hospital context because those responsibilities are primarily fulfilled by other services of the National Health System. Some participants (DE, IT, NO) expressed uncertainty about which part of the organization (the entire hospital or individual departments/units) was to be evaluated, as the degree of fulfilment could vary across departments.

Missing or N/A responses were observed for almost all indicators (see supplementary file 2 for indicators with high missing values or N/A responses (≥ 50%) for each participating hospital). For example, half of the participants in the hospitals in AT, DE, IT-B, and RS did not provide responses for whether automated phone systems have a clear option to repeat menu items (indicator 4.1.7). Similarly, indicators related to communication guidelines (indicator 5.1.2a–e) had ≥ 50% missing values or N/A responses in the hospitals in AT, DE, and IT-B. The same was evident in the hospitals in AT, DE, and NO for the indicator about pre-testing digital services (indicator 5.3.3), in the hospitals in AT, IT-A, and IT-B for the indicator about HL interventions for hard-to-reach groups (indicator 8.1.3), and in the hospitals in DE, IT-A, and IT-B for the indicator about public reporting of HL activities (indicator 8.2.1).

Results from the individual assessments were discussed at the joint assessment with the aim of agreeing on strengths and weaknesses concerning OHL either of the organization or of the organizational unit. In Italian, Norwegian and Serbian hospitals, several indicators with different responses from the individual assessments were observed. Experience from the joint assessment showed that different viewpoints were due to the respondents representing different departments and functions. Following discussion, the assessment teams were usually able to reach agreement on the rating of indicators. Therefore, results from the joint assessment partly differed from the individual assessments. In the German hospital it was reported that relevant aspects that were not originally covered in the tool were identified during the joint assessment.

#### Integration

Regarding integration, (i.e., the level of system change needed to integrate a new process into an existing infrastructure), the internal coordinators within the hospitals and/or participants reported that the tool was suitable for assessing OHL. The tool and the self-assessment process led to awareness and valuable insight when it comes to OHL and stimulated reflections and discussions concerning improving OHL in all hospitals.

IT reported that the joint assessment was also an opportunity for participants to become aware of certain actions already planned within the hospital. The Serbian hospital-internal coordinator reported: *’By filling in the tool and later with the joint assessment*,* participants became aware of certain problems*,* but also how they can work to solve those problems. The different opinions of the participants have opened a space for thinking about how to improve work and relationships with patients. It is also important for the hospital to have an insight into its own shortcomings so that they can implement measures for improvement’ (hospital-internal coordinator*,* RS)*.

Based on the individual and joint assessments, the Czech project team identified development priorities, which were then submitted to and approved by the hospital management. To maintain the sustainability of the project and to ensure the implementation of proposed interventions, the hospital management decided to formulate an action plan to be implemented within two years. Interventions of the action plan focus on the education of the public and especially children and students (e.g., visits to primary and secondary schools and in the hospital), improving the communication between the hospital staff and patients in the form of audits, training and regular education, launching a patient portal, and more effective transfer of electronic documentation between doctors and providers.

Participants from the Austrian hospital reported that it remained open after the joint assessment who would take responsibility for further actions.

### OHL strengths and areas for improvement

**Fig. 3:**
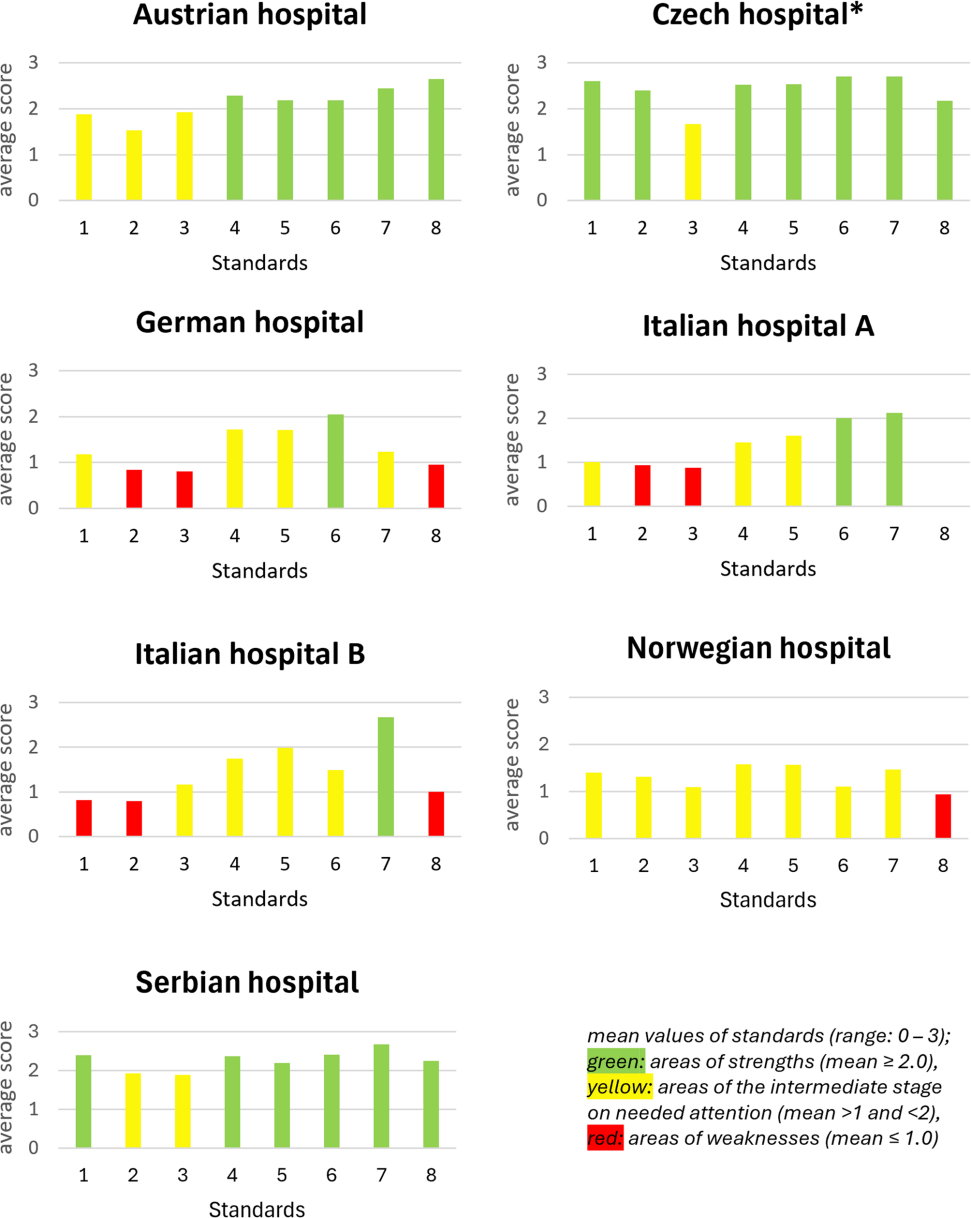
The average degree of fulfilment for Standards 1–8 for each hospital in six countries

Exploring the OHL strengths and weaknesses, most standards had a high degree of fulfilment in the Czech and Serbian hospitals. Standards 6 (*Promote personal HL of patients and relatives after discharge*) and 7 (*Promote personal HL of staff with regard to occupational risks and personal lifestyles*) obtained a high degree of fulfilment in five out of seven hospitals. Low fulfilment was observed for Standards 2 (*Develop documents*,* materials and services with stakeholders in a participatory manner*), 3 (*Enable and train staff for personal HL and OHL*), and 8 (*The organization contributes to the improvement of personal HL of the local population*) in several hospitals (Fig. 3).

**Fig. 4:**
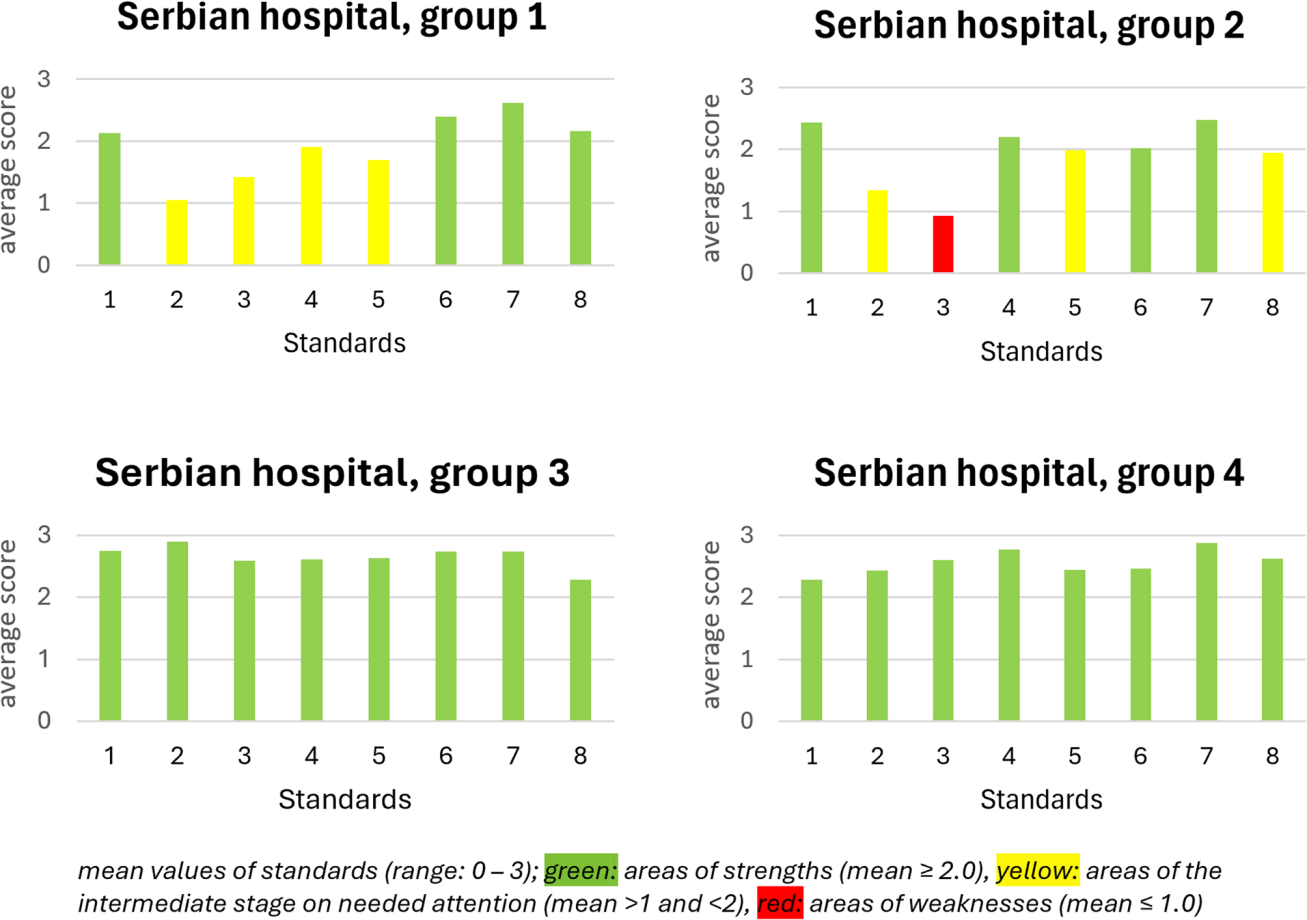
The average degree of fulfilment for Standard 1–8 for the Serbian joint assessment groups

In the Serbian hospital the joint assessment was conducted in four groups. In groups 3 and 4, where the participants were nurses and medical doctors, respectively, all standards obtained a high degree of fulfilment. Other therapeutic professions (group 1) assessed Standards 1, 6, 7, and 8 as highly fulfilled, whereas the others could be considered at the intermediate level (medium fulfillment). The managerial capacities (group 2) identified Standard 3 as an area of weakness (low fulfillment), Standards 2, 5, and 8 at the intermediate level, and Standards 1, 4, 6, and 7 as highly fulfilled (Fig. 4).

## Discussion

The aim of this article is to present the experiences of piloting the OHL-Hos in seven hospitals in six European countries. To that effect, the feasibility of the tool and the self-assessment process were explored. In summary, in all hospitals it was found possible to self-assess the status of OHL using the OHL-Hos following the self-assessment process, although partly with adjustments. Also, in all hospitals, OHL strengths and areas for improvement could be identified and agreed upon. However, the OHL-Hos, being a comprehensive tool with eight standards, 21 sub-standards and 141 indicators, and the self-assessment process consisting of several steps, posed challenges to the self-assessment teams and the internal coordinators within the hospitals. This led to recommendations for the improvement of the tool and the self-assessment process.

The *acceptability* of the tool was reflected by the fact that a mandate was obtained from the management of all hospitals, involving a multidisciplinary assessment team and finalizing the self-assessment process. The joint assessment, in which the members of the assessment team jointly agree on indicator ratings and areas of improvement, was highly valued by the participants. In the joint assessment results from the individual assessment were presented and discussed and it was possible for the assessment team to agree on a different rating after discussing the different perspectives, clarifying meanings or providing additional information of relevance for rating the indicator. The use of such co-creational strategies for OHL assessments was suggested by Palumbo [28], ensuring that they are not treated as mere management tools. Another comprehensive tool, the Organizational Health Literacy Responsiveness tool (Org-HLR) [29] also relies on joint assessment. The inclusion of patients in the OHL self-assessment is considered a facilitating factor [15]. In our study, patient representatives should have been included, but this was only achieved in two hospitals. Including patient representatives could help identify gaps that hospital staff may overlook and promote patient-centered care. The complexity and the length of the OHL-Hos as well as the time needed to complete the self-assessment process led to dissatisfaction and decrease in acceptability.

Although there were some differences in the self-assessment process in the hospitals (number of participants involved, pre-collection of individual assessment results before the joint assessment, grouping the participants for the joint assessment), all participating countries were able to *implement* the self-assessment successfully. The hospital-internal coordinators who offered an introductory workshop considered these workshops as very important for the successful implementation of the OHL self-assessment process. Conducting an introductory workshop was also identified as a facilitator in other studies [15]. The self-assessment process allowed hospitals sufficient flexibility to adapt to their needs and resources, as demonstrated by the specific approaches taken by different countries, which we consider an advantage.

With respect to *practicality*, the OHL-Hos was considered lengthy with complex wording, and its format was considered unsatisfactory by some participants who would have preferred an online option. To increase practicality, shortening an instrument/tool is a common process in health and health services research [30, 31]. Some indicators and even entire sub-standards need specific knowledge depending on the respondent’s position within the hospital (e.g. some parts need managerial insights, some need experience with patient contact), and could not be answered or were not of relevance to some (groups of) participants. This leads to the suggestion that certain indicators of the tool may only be answered by certain professional or managerial/technical groups when assessing OHL in hospitals. Clarifying the scope of the assessment, e.g., a ward or department or the entire hospital, and communicating it to the assessment team may also be considered.

Our study showed that *integrating* an OHL self-assessment process can be conducted based on existing structures and resources with the hospital and without enormous efforts or systems changes. This assumption is supported by literature [10, 21]. Although supporting the implementation of OHL interventions based on the assessment was outside the scope of this pilot study, the decision to develop and implement an action plan by the Czech hospital illustrates the possibility of such a process. Feedback from internal coordinators within the hospitals shows that the delegation of the responsibilities for the implementation process should also be defined as part of the self-assessment process.

The self-assessment process by means of the OHL-Hos can lead to the identification of *strengths and areas for improvement of OHL*, as the joint assessment promotes decision-making based on a consensus, even in the case of different ratings of indicators by individual team members. Results from different professional groups from the Serbian hospital indicate that the composition of the assessment team influences the results. This shows the importance of the professional perspective from which results are obtained when interpreting the results. The involvement of a multidisciplinary and inter-hierarchical team in the self-assessment process and bringing together different perspectives in a joint assessment, as suggested by the OHL-Hos instructions, is thus considered highly relevant.

We refrained from *benchmarking* as the results stem from only one hospital in five countries and two in one country. In addition, the selection of hospitals was based on a convenience sample and was opportunistic. Yet we would like to refer to the research question on benchmarking more theoretically. The possibility of creating self-assessments based on standardized response options makes the OHL-Hos basically suitable for future benchmarking. In terms of content, however, such a process must be monitored for usefulness and accuracy. The tool involves quantitative and qualitative indicators, so not all indicators are numerically comparably in terms of strict quantitative measures. Both types of indicators have their respective values and importance, but benchmarking could be problematic for some indicators. Yet, benchmarking across hospitals can be challenging for two reasons. Firstly, implementation can vary; for instance, hospitals may apply the tool differently, making direct comparisons difficult. Secondly, context-specific factors, such as local healthcare structures, cultures, and policies influence how indicators are understood and rated. In the case of international benchmarking, in addition, the impact of different health care systems needs to be taken into account. Likewise, in the case of national benchmarking, regional differences should be considered.

### Limitations

We acknowledge that this study has several limitations. For this feasibility study only one hospital in each country, except for IT with two hospitals, was recruited. Hospital selection was intentionally opportunistic in order to pilot the tool and the process. We cannot generalize experiences and results to other hospitals within a country nor among an international group of hospitals. In our study, hospital-internal coordinators selected and recruited participants for the self-assessment process. Their reasons and motivation for participating may have influenced results. Furthermore, there is a potential bias stemming from hospital-internal coordinators selecting participants, particularly regarding power hierarchies, the representation of different departments, and the possibility that coordinators might have selected individuals who are either particularly positively or negatively engaged with HL or OHL. Not all professional groups were represented in every hospital, and for most hospitals specific units were selected for the OHL self-assessment. This uneven distribution limits the generalizability of the findings across professions and hospitals (also within hospitals), as certain perspectives may be underrepresented or missing entirely. Patient representatives should have been included as their perspective might differ from those of professionals, but unfortunately this was only achieved in two hospitals. The statistical analysis of the results is only descriptive and a high number of missing values for some indicators were recorded. The role and impact of missing values should be more systematically explored in future studies. But despite these limitations we are convinced that the valuable insights gained, and lessons learned by the seven hospitals are important and meaningful for recommending improvements to the OHL-Hos and the self-assessment process.

### Recommendations for the OHL-Hos

- A shortened version of the tool should be developed and applied in its entire version.
- A shortened version of the tool should use simple/clear language.
- Wording for the response categories that is suitable for international use should be used.
- As the OHL-Hos is a comprehensive and lengthy tool, it is recommended that it is used in a modular format, i.e. by conducting an assessment of selected standards or sub-standards. This allows for an in-depth assessment of the selected standards or sub-standards.

### Recommendations for the self-assessment process

- Conduct an introductory/orientation workshop for the assessment team.
- Clearly communicate the scope of the assessment (unit or the whole organization) to the assessment team.
- As some staff may only be able to assess individual standards or sub-standards, but not all standards/sub-standards, appropriate guidance should be provided on who should complete which part of the tool.
- Pre-selecting parts (e.g. by the hospital-internal coordinator) to be completed by participants to reduce missing values and save participants’ time by not having to go through parts they cannot answer and/or are not relevant for them.
- Responsibilities for further improvement of OHL in the hospital should be defined.

To support organizations using OHL assessment tools and subsequently to make the necessary further improvements, more supportive regulations, policies and resources are needed. As a prerequisite for this, more attention from health care policy and administration is needed [17]. Some countries already support the implementation of OHL on the political level: AT [32, 33], Australia [34], Canada [35, 36], DE [37, 38], New Zealand [39], NO [40], Scotland [41] and the United States [12, 42–45].

One way of facilitating OHL assessments and interventions is the inclusion of OHL standards or indicators in accreditation systems [8]. Another aspect is to make OHL more legally anchored in the responsibilities of health care organizations and in the education and training of health care professionals. Health care organizations can adapt their policies, structures and processes to mitigate the effect of HL challenges and thereby enhance their OHL [46–48]. OHL assessments can support the transformation towards becoming a health literate health care organization, provided that the assessment process and the used tool are feasible and perceived as relevant [15, 18, 28].

## Conclusions

The OHL-Hos was successfully piloted in seven hospitals across six European countries, identifying strengths and areas for improvement in OHL. The comprehensive nature of the tool and the self-assessment process posed challenges, leading to recommendations for simplifying and improving both the tool and the process. The process was generally accepted, with multidisciplinary and multi-hierarchical teams playing crucial roles. Joint assessments were particularly appreciated for fostering consensus. Certain indicators required specific knowledge, suggesting that different professional groups should address relevant parts of the assessment. Flexibility in the self-assessment process and the importance of introductory workshops were noted as key factors for successful implementation. Further validation is needed. Recommendations include developing a shortened version of the tool with simple language. In case the OHL-Hos is used, a modular approach is recommended. The existence of feasible tools and processes for their application is an essential prerequisite for the introduction and sustainable implementation of OHL in hospitals.

## Supplementary Information

Supplementary Material 1.

Supplementary Material 2.

## Data Availability

The OHL-Hos is available in Czech, German, Italian, Norwegian, and Serbian upon request at m-pohl@goeg.at. The English version can be found at https://m-pohl.net/ReferencesOHL. To protect the privacy of participants and hospitals, the data supporting the results of this study are not publicly available.

## Abbreviations

AT: Austria
DE: Germany
CZ: Czech Republic
HL: Health literacy
IT: Italy
IT-A: Hospital A in Italy
IT-B: Hospital B in Italy
M-POHL: WHO Action Network on Measuring Population and Organizational Health Literacy
N/A: Not applicable
NO: Norway
OHL: Organizational health literacy
OHL-Hos: International Self-Assessment Tool for Organizational Health Literacy (Responsiveness) of Hospitals
Org-HLR: Organisational Health Literacy Responsiveness tool
Sd: Standard deviation
RS: Serbia
V-HLO-I: Vienna tool of Health-Literate Hospitals and Healthcare Organizations
