# Supplementary material for: Assessing organizational health literacy in hospitals by using the International Self-Assessment Tool for Organizational Health Literacy of Hospitals – a feasibility study in six European countries
Source: BMC Health Serv Res. 2025 Oct 1;25:1265. doi: 10.1186/s12913-025-13367-4 (PMC12487070; doi:10.1186/s12913-025-13367-4)
Supplement: Supplementary file 1 — Supplementary Material 1. [file 12913_2025_13367_MOESM1_ESM.docx]

### OHL-Hos self-assessment

### Semi-structured interview questionnaire

#### 1 Individual assessments

1.a Please describe the participants of the individual assessment by the following groups, indicating the number of people of each group

| ***Description – participants from:*** | ***No. of persons*** |
| --- | --- |
| Management |  |
| Quality management |  |
| Health promotion |  |
| Human resource development |  |
| Medicine |  |
| Nursing |  |
| Therapeutic professions |  |
| Building services engineering/maintenance |  |
| Patient-ombudsman/woman, self-help and patient representatives. |  |
| Communications/spokesperson |  |
| Others, please indicate |  |
| Others, please indicate |  |
| Others, please indicate |  |
| Others, please indicate |  |

1.b Were individual assessments conducted according to the study protocol?

| Yes/no |
| --- |

and if not, what was different?

| (Free text) |
| --- |

1.c How long did the individual assessments take on average?

Provide in average length in minutes: _____________

Were the duration and efforts to complete the individual assessment acceptable – please describe:

(Free text)

1.d Were there any difficulties experienced in individual assessments – please describe:

| (Free text) |
| --- |

1.e Other issues and comments

| (Free text) |
| --- |

#### 2. Joint assessment

2.a Describe the participants of the joint assessment

| ***Description – participants from:*** | ***No. of persons*** |
| --- | --- |
| Management |  |
| Quality management |  |
| Health promotion |  |
| Human resource development |  |
| Medicine |  |
| Nursing |  |
| Therapeutic professions |  |
| Building services engineering/maintenance |  |
| Patient-ombudsman/woman, self-help and patient representatives. |  |
| Communications/spokesperson |  |
| Others, please indicate |  |
| Others, please indicate |  |
| Others, please indicate |  |
| Others, please indicate |  |

2.b Describe the process of the joint assessment

Was the joint assessment conducted according to the study protocol?

| Yes/no |
| --- |

and if not, what was different?

| (Free text) |
| --- |

Who moderated the joint assessment? (e.g., the person responsible for the individual assessments, the national coordinator, external moderator, etc.?)

| (Free text) |
| --- |

2.c How long did the joint assessment last?

Provide in average length in minutes: _____________

Were the duration and efforts considered worthwhile and relevant by the participants?

| (Free text) |
| --- |

2.d What were the main discussion points at the joint assessment?

| (Free text) |
| --- |

Was there consensus on discussion points?

| (Free text) |
| --- |

What were the areas of disagreement?

| (Free text) |
| --- |

2.e Were there any issues that remained open after the joint assessment?

| (Free text) |
| --- |

2.f Did the joint assessment produce a diagnosis of the strength and weaknesses concerning organizational health literacy of the institution or of the specific unit? Please describe in detail:

| (Free text) |
| --- |

2.g Difficulties experienced with the process of the joint assessment (that were not yet reported) – please describe in detail:

| (Free text) |
| --- |

2.h Other issues and comments

| (Free text) |
| --- |

#### 3. Identifying difficulties and suggestions for improvement of the OHL self-assessment instrument

Using individual assessment sheets, the assessment summary sheets and the results from the joint assessment, please…

3.a …sum up the group’s feedback at indicator level and indicate problematic indicators.

| (Free text) |
| --- |

3.b …report on suggestions for improvement for problematic indicators.

| (Free text) |
| --- |

3.c …summarize the group’s consensus regarding the overall utility of the OHL self-assessment instrument and the assessment process

| (Free text) |
| --- |

3.d …report on: Is the wording considered clear and comprehensive?

| (yes/no) |
| --- |

If not, which wording / text parts need improvement?

| (Free text) |
| --- |

3.e …report on: Is the assessment scale appropriate?

| (yes/no) |
| --- |

If not, which wording / text parts need improvement?

| (Free text) |
| --- |

3.f Other issues and comments

| (Free text) |
| --- |
