# Supplementary material for: Assessing organizational health literacy in hospitals by using the International Self-Assessment Tool for Organizational Health Literacy of Hospitals – a feasibility study in six European countries
Source: BMC Health Serv Res. 2025 Oct 1;25:1265. doi: 10.1186/s12913-025-13367-4 (PMC12487070; doi:10.1186/s12913-025-13367-4)
Supplement: Supplementary file 2 — Supplementary Material 2. [file 12913_2025_13367_MOESM2_ESM.docx]

**Supplementary information 2**

Indicators (marked with an x) with high missing values and N/A responses (≥ 50%) for each participating hospital

| **Indicator** | **Wording** | **AT** | **CZ** | **DE** | **ITa** | **ITb** | **NO****^i^** | **RS** |
| --- | --- | --- | --- | --- | --- | --- | --- | --- |
| 1.1.4 | The management of the organization serves on oversight committees for organizational health literacy. | X |  |  |  |  |  |  |
| 1.2.1 | Policy documents such as the mission statement, goals, and policies, explicitly define health literacy as an organizational priority. |  |  | X |  |  |  |  |
| 1.2.3 | Financial resources for promoting organizational health literacy are defined and allocated in business / operational plans. |  |  | X |  |  | X |  |
| 1.2.4 | Qualified personnel for promoting organizational health literacy is defined and allocated in business / operational plans. |  |  | X |  |  |  |  |
| 1.2.6 | Organizational health literacy is promoted by all organizational units and policies. *(E. g. in the units and policies for quality management, health promotion, risk management, human resource management, facility management)* |  |  | X |  |  |  |  |
| 1.3.1a) | Organizational health literacy is integrated into the existing quality management system by definition of criteria and indicators. | X |  | X |  |  |  |  |
| 1.3.1b) | Organizational health literacy is integrated into the existing quality management system by regular assessment. |  |  | X |  |  |  |  |
| 1.3.1c) | Organizational health literacy is integrated into the existing quality management system by monitoring and improving of activities. | X |  | X |  |  |  |  |
| 1.3.2 | Patient surveys include questions about the quality of information and communication. *(E. g. comprehensibility of information provided)* |  |  | X |  |  |  |  |
| 1.3.3 | Staff surveys include questions about the quality of information and communication. *(E. g. comprehensibility of information about occupational health and safety)* |  |  | X |  |  |  |  |
| 1.3.5 | Staff surveys use clear, everyday words and phrases. | X |  | X |  |  |  |  |
| 1.3.6 | Patient health literacy is part of performance measurement of the organization. |  |  | X |  |  |  |  |
| 1.3.7 | The organization uses “mystery patients” (🡪 glossary) or “walking interviews” (🡪 glossary) to assess how easy it is for patients/visitors to navigate the organization. |  |  |  |  |  | X | X |
| 1.3.8 | The organization uses “mystery patients” to assess the quality of communication with and the quality of information for patients (verbal, written, visual). |  |  |  |  |  | X | X |
| 2.1.2 | The navigation system of the organization is tested by patients and is improved following the outcomes. |  |  | X |  |  |  |  |
| 2.1.3 | Guidelines and procedures for staff on patient communication are developed and tested not only with representatives of staff but also of patients. *(E.g. persons with limited reading skills, members of specific ethnic groups.)* |  |  | X |  |  |  |  |
| 2.1.4 | (Former) Patients or trained simulated patients are involved in the training of staff in order to provide feedback on staff's oral communication skills. |  |  | X |  |  |  |  |
| 2.1.5 | The organization implements mechanisms and procedures to enable feedback and complaints by patients concerning comprehensibility of documents, materials and services. |  |  | X |  |  |  |  |
| 3.1.1 | Documents such as job descriptions, selection criteria for applicants, staff development plans etc. include health literacy as a main competence. |  |  | X |  |  |  |  |
| 3.1.5 | Internal health literacy experts serve as role models, mentors and teachers of health literacy competences to others. |  |  |  | X | X |  |  |
| 3.1.6e) | Staff are offered trainings with regard to: Effective risk communication as the basis for informed patient consent on medical treatment. |  |  | X |  |  |  |  |
| 3.1.6f) | Staff are offered trainings with regard to:Motivational interviewing (🡪 glossary). | X |  |  |  |  |  |  |
| 3.1.6g) | Staff are offered trainings with regard to:Use of written and audio-visual materials to support communication *(E. g. decision aids).* |  |  | X |  |  |  |  |
| 3.1.6h) | Staff are offered trainings with regard to: Basic knowledge on designing easy-to-understand print materials. |  | X |  |  |  |  |  |
| 3.1.6i) | Staff are offered trainings with regard to: When and how to use an interpreter (🡪 glossary), and how to effectively collaborate with interpreters. |  | X |  |  |  |  |  |
| 4.1.7 | If there is an automated phone system, there is a clear option to repeat menu items. | X |  | X |  | X |  | X |
| 4.1.8 | Telephone communication is available in most native languages of patients. |  |  | X |  |  |  |  |
| 4.1.9 | People at a hotline or an information desk are qualified to adequately answer patient enquiries. |  |  | X |  |  |  |  |
| 4.2.3 | The healthcare organization negotiates with local transportation services to assist patients by displaying adequate signage, clear announcements, and location information at public transportation stations. | X |  | X |  |  |  |  |
| 4.3.3 | Maps clearly indicate the individual's location in the hospital through easy-to-understand symbols or "You are here" signage. |  |  |  |  | X |  |  |
| 4.3.12 | Signage is available between buildings if the organization contains multiple buildings. |  |  |  |  | X |  |  |
| 4.4.1 | Patients are informed about deductibles or other costs for treatment or services in advance. *(E.g. on the website and by telephone enquiry*) |  |  | X |  |  |  |  |
| 4.4.2 | Patients are informed about their patient rights. |  |  | X |  |  |  |  |
| 4.4.3 | A physical or virtual patient information center comprising free health information is available. |  |  | X |  |  |  |  |
| 5.1.2a) | Communication guidelines consider the diverse needs of different patient groups: Patients of different linguistic, ethnic and cultural backgrounds. | X |  | X |  | X |  |  |
| 5.1.2b) | Communication guidelines consider the diverse needs of different patient groups: Patients with impaired visual capabilities. | X |  | X |  | X |  |  |
| 5.1.2c) | Communication guidelines consider the diverse needs of different patient groups: Patients with impaired hearing capabilities. | X |  | X |  | X |  |  |
| 5.1.2d) | Communication guidelines consider the diverse needs of different patient groups: Patients with impaired intellectual capabilities. | X |  | X |  | X |  |  |
| 5.1.2e) | Communication guidelines consider the diverse needs of different patient groups: Patients with the need to involve relatives/caregivers. | X |  | X |  | X |  |  |
| 5.2.4 | Written and audio-visual materials are revised periodically to ensure best quality and accuracy of information *(e.g. based upon current evidence).* Materials include a statement of last update and the information source so that the quality of the original information source can be assessed independently. |  |  | X |  |  |  |  |
| 5.3.1 | Guidelines for the quality and distribution of digital services and new media are used to support communication and information transfer. | X |  | X |  |  |  |  |
| 5.3.2 | All digital services and new media which are available via online portals, app download centers etc. are technically correct, easy-to-understand, contain action-oriented information and are adequate for target groups. |  |  | X |  |  |  |  |
| 5.3.3 | Digital services and new media are pre-tested with representatives of target groups and patients before distribution. | X |  | X |  |  | X |  |
| 5.4.4 | Patients are informed about professional translation services routinely at admission and on demand. |  |  |  |  |  |  | X |
| 5.4.5 | If needed, professional translation services are always available for medical examinations and consultations with clinical staff and also provide assistance in completing forms or documents. *(E.g. in house interpreters, telephone / video interpreting)* |  |  |  |  |  |  | X |
| 5.4.6 | There is a coordination office for the provision and scheduling of translation services in native language. |  |  |  |  |  |  | X |
| 5.4.7 | Interpreters / translators are specifically qualified / certified in inter-cultural medical translation. *(E.g. language certificates, letter of recommendation).* |  |  |  |  |  | X | X |
| 5.4.8 | All interpreters / translators are trained to use clear, everyday words and phrases. |  |  |  |  |  |  | X |
| 5.4.9 | Guidelines for reporting, documenting and processing problems and complaints with regard to translation services are available. Problems are monitored and improvement measures are implemented. |  |  | X |  |  |  | X |
| 5.5.1 | The organization considers communication errors as adverse events and reacts by analyzing the origin of detected errors and by improving communication processes. |  | X | X |  |  |  |  |
| 5.5.2 | A reporting and performance monitoring system for communication errors is available. |  | X | X |  |  |  |  |
| 5.5.3 | Feedback from patients regarding patient safety, hospital hygiene etc. are routinely included in risk management. *(E.g. patient surveys, feedback forms, patient complaints)* |  | X | X |  |  |  |  |
| 5.5.4 | A list of processes and procedures is available, that pose a higher risk to patients, and therefore require a heightened level of assurance to ensure that patients have fully understood the information provided.  *(E.g. patient communication about diagnosis, therapies, consent forms, filling in forms, preparation for surgeries, transferals)* | X | X |  |  |  |  |  |
| 5.5.5 | There exist specific guidelines and staff trainings on communication in situations that pose a higher risk to patients *(E.g. breaking bad news, new therapies, preparation for surgeries)* are available. |  | X |  |  |  |  |  |
| 5.5.6 | Taking medication is explained in detail (including clarification that medicines prescribed in the hospital can differ from those distributed in pharmacies). |  | X |  |  |  |  |  |
| 5.5.7 | Aids such as pill boxes, charts, etc. are used to increase comprehensibility of taking medicines correctly. |  | X | X |  |  |  |  |
| 5.5.8 | The organization's emergency plan contains easy-to-use information for patients regarding evacuation. It also addresses people who are illiterate, with hearing or visual impairment and / or different intellectual capabilities, and other vulnerable types of patients. |  | X |  |  |  |  |  |
| 6.3.10 | During discharge, Patients routinely receive contact details of relevant patient advocates and patients' ombudspersons. *(E.g. in case of complications or complaints)*. |  |  | X |  |  |  |  |
| 7.1.3 | Performance reviews include status information on occupational health and safety, and on how staff can maintain their health. |  |  | X |  |  |  |  |
| 7.1.8 | The organization provides measures for prevention or self-management of chronic conditions of staff. |  |  |  |  | X |  |  |
| 8.1.1 | The organization provides evidence-based and non-commercial information about relevant health topics issues to the local community it serves. *(E.g. through health fairs, public lectures)*. |  |  |  | X | X |  |  |
| 8.1.2 | The organization drives health education and promotion initiatives to build skills for health literacy in the local population. *(E.g. by organizing workshops on workplace health promotion in local companies, or facilitating guided tours to the hospital for students from local schools)* |  |  |  | X | X |  |  |
| 8.1.3 | The organization conducts interventions to improve health literacy of hard-to-reach patients / citizen groups at the local level. *(E.g. interactive meetings with socio-economically disadvantaged groups or migrant communities)* | X |  |  | X | X |  |  |
| 8.2.1 | Health literacy activities and outcomes are part of the organization's public reporting. |  |  | X | X | X |  |  |
| 8.2.2 | The organization communicates experiences with organizational health literacy practices via publications, presentations, and other media. |  |  | X | X | X |  |  |
| 8.2.3 | The organization participates in health literacy research and development projects. |  |  | X | X |  |  |  |
| 8.2.4 | The organization contributes to wider (policy) goals or action plans in the field of health literacy. |  |  | X | X |  |  |  |
| 8.2.5 | The organization offers health literacy best practices for the professional training of doctors, nurses, and other relevant professional groups also outside of the organization. |  |  | X | X |  |  |  |

^i^Some respondents were not offered indicators of sub-standard 1.1. These are not included in the table.
